# Effects of working from home on lifestyle behaviors and mental health during the COVID-19 pandemic: A survey study

**DOI:** 10.1371/journal.pone.0300812

**Published:** 2024-04-01

**Authors:** Egeria Scoditti, Antonella Bodini, Saverio Sabina, Carlo Giacomo Leo, Pierpaolo Mincarone, Antonella Rissotto, Stanislao Fusco, Roberto Guarino, Giuseppe Ponzini, Maria Rosaria Tumolo, Nicola Magnavita, Giovanni Luigi Tripepi, Sergio Garbarino

**Affiliations:** 1 Institute of Clinical Physiology (IFC), National Research Council (CNR), Lecce, Italy; 2 Institute for Applied Mathematics and Information Technologies "E. Magenes" (IMATI), National Research Council (CNR), Milano, Italy; 3 Institute for Research on Population and Social Policies (IRPPS), National Research Council (CNR), Research Unit of Brindisi, Brindisi, Italy; 4 Training and Welfare Unit, National Research Council (CNR), Rome, Italy; 5 Institute for Research on Population and Social Policies (IRPPS), National Research Council (CNR), Research Unit of Brindisi, Brindisi, Italy; 6 Department of Biological and Environmental Sciences and Technology (DISTEBA), University of Salento, Lecce, Italy; 7 Post-graduate School of Occupational Health, Università Cattolica del Sacro Cuore, Rome, Italy; 8 Institute of Clinical Physiology (IFC), National Research Council (CNR), Reggio Calabria, Italy; 9 Department of Neurosciences, Rehabilitation, Ophthalmology, Genetics and Maternal/Child Sciences (DINOGMI), University of Genoa, Genoa, Italy; University of Udine: Universita degli Studi di Udine, ITALY

## Abstract

The COVID-19 outbreak has led to relevant changes in everyday life worldwide. One of these changes has been a rapid transition to and an increasing implementation of working from home (WH) modality. This study aimed to evaluate the impact of mandatory WH during the COVID-19 pandemic on lifestyle behaviors, Mediterranean diet adherence, body weight, and depression. An online cross-sectional survey was conducted in the early 2022 at the National Research Council of Italy using ad hoc questions and validated scales collecting information on physical activity, sedentary behavior, hobbies/pastimes, dietary habits including adherence to the Mediterranean diet, body weight, and depression during WH compared with before WH. 748 respondents were included in the study. An increased sedentary lifetime was reported by 48% of respondents; however, the subsample of workers who previously performed moderate physical activity intensified this activity. Body weight gain during WH was self-reported in 39.9% of respondents. Mediterranean diet adherence increased (p≪0.001) during WH compared with before WH. The average level of mental health did not record an overall variation; however, the proportion of subjects with mild and moderate depression increased (p = 0.006), while workers who reported values indicative of depression before the transition declared an improvement. These findings highlight health-related impact of WH during the COVID-19 pandemic that may inform future strategies and policies to improve employees’ health and well-being.

## Introduction

The COVID-19 outbreak has led to sudden and unexpected changes in life and work patterns worldwide [1, 2]. In response to the COVID-19 pandemic, governments and organizations worldwide promoted the adoption of working from home (WH) (also referred to as teleworking or remote working) [3], a conception of work performed in places, like home, other than a traditional office space and centered on the flexibility and autonomy of the work assisted by information and communication technological tools [4]. WH was mandatory during home confinements (in Italy from March 9 to May 3, 2020). Subsequent to the gradual relaxation of the confinement measures in accordance with the evolving epidemiological situation, WH was still strongly encouraged, whenever feasible, to contain the spread of the SARS-CoV-2 virus [3, 5]. The WH modality in itself might have consequences not only for self-management, work performance, work-family balance, and social interactions, but also for lifestyle behaviors and health outcomes as documented by studies conducted before the COVID-19 pandemic [6–8]. The COVID-19 pandemic represents a useful and specific context for studying the impact of an unanticipated and unstructured WH on individuals’ adaptation to a new work environment in the face of previously limited or no experience [7–9]. During the CODIV-19 pandemic and the associated crisis situation, the new work practices such as WH produced significant consequences for workers’ productivity, engagement, physical and mental health and wellbeing [10–17]. In particular, either an increase [13, 14] or a decrease [9, 12] of anxiety or depressive symptoms, along with other mental health issues, was found subsequent to the implementation of WH under the COVID-19 pandemic. Nonetheless, the effects of WH on lifestyle behaviors such as diet, including the adherence to the Mediterranean diet, physical activity and smoking habit, as well as on body weight [18–21], which are risk factors responsible for a significant proportion of disease burden and mortality worldwide [22, 23], have not been extensively studied.

Exploring the effect of WH on lifestyle behaviors and health outcomes would contribute to better understand its potential benefits and risks for health, as a result of (mal)adaptive responses and coping strategies adopted by individuals under an unprecedented stressful circumstance linked to the COVID-19 pandemic and the new work reorganization demands. This knowledge would inform future studies, ultimately providing avenues for better defining remote working and public health policies. This could be important given that some workplaces are considering implementing the WH modalities permanently, beyond the COVID-19 pandemic period [24].

Considering that the evidence on the health impacts of WH during the COVID-19 pandemic is scarce, the objective of this study was to investigate, in the largest public research organization of Italy, the National Research Council (CNR), the impact of the introduction of WH modalities during the COVID-19 pandemic on key health-related parameters such as lifestyle behaviors (dietary habit including adherence to the Mediterranean diet, physical activity, sedentary behavior), and health outcomes (body weight, and depression). To accomplish this objective, we conducted an online survey among non-essential workers who had transitioned to WH for 18 months (since its first implementation in March 2020), assessing current and retrospective (pre- COVID-19 pandemic) ratings of the analyzed health parameters. Since adopting a cross-sectional design did not allow us to separate the impacts of the change in work activity from those induced by the COVID-19 pandemic, we formulated some a priori hypotheses, based on literature data: the first, that the fear of the pandemic and the occupational stress caused by it were not particularly relevant for workers who did not have unprotected contact with infectious patients, as was demonstrated by studies conducted on healthcare workers during the first COVID-19 pandemic phase [25, 26]. The second, that WH can have significant effects on workers’ health, as shown by studies conducted before the COVID-19 pandemic [6, 8]. The third, that a pandemic period determines such a significant change in life and work habits that it can be easily recorded with a cross-sectional study with a retrospective assessment.

## Materials and methods

### Study design and population

We conducted a cross-sectional online survey among permanent workers at the CNR. CNR work organization and study population were described in a previous published manuscript [27]. Before the COVID-19 pandemic, the CNR had not yet introduced WH and only a small percentage of workers (around 5%) was working from home.

The data reported here were part of a wider research project (“WorkInCovid” Study) concerning the impact of WH, as a consequence of the COVID-19 pandemic, on health, wellbeing, work experience and quality of life of full-time employees of the CNR. The development and implementation of the online survey were previously described in detail in [27] and are here briefly reported. Results from different analyses were recently published [27].

### Survey

The survey begun on 12 January 2022 and was closed on 9 March 2022. Data were collected using the LimeSurvey open source tool (Community Edition version 3.26.1). After authorization of the CNR General Manager, the invitation to participate in the survey was sent by e-mail to the CNR mailing list including all fixed-term or permanent workers. Workers who were hired after 1 June 2019 were excluded from the study as their ordinary work experience was too limited to be included in the study. Up to 3 follow-up emails were sent to remind employees to complete the survey. Before the official launch of the study, the survey questions were pretested for clarity, completeness, privacy issues, and technical aspects in a pilot phase conducted in a small group of volunteers among the CNR employees and were refined accordingly.

In brief, the survey, which took approximately 40 min to complete, included standard validated questionnaires and ad hoc questions specifically developed by the authors for this survey. The first section, which was the only mandatory, assessed: a) sociodemographic data: age, gender, educational level, professional profile, marital status, number of children, hometown and home dimensions, residence (Northern Italy, Southern Italy, Central Italy, islands), availability of private outdoor area at home, engagement in family care; and contacts with SARS-CoV-2 infected subjects (“Yes”, “No”); b) work-related characteristics: number of days required to work at workplace during the WH period, home-to-work commuting time and means of transport before WH, type of workroom at home during WH, frequency of workstation sharing; participants were asked to rate their perceived impact of WH on work experience (not assessed in the present work, see [27]); c) participants were also asked to report their habit of practicing physical activity during the WH period i.e. walking, moderate-intensity physical activity, and vigorous-intensity physical activity (as defined in the International Physical Activity Questionnaire, IPAQ [28]), sedentary behavior (sitting time) and engagement in hobbies or pastimes, and to rate changes in these habits during WH in terms of time spent as “Much increased”, “Increased”, “Unchanged”, “Decreased”, “Much decreased”.

Another section collected information on: body weight and height (for the calculation of body mass index, BMI) and self-reported weight change during WH (“No”, “Yes, decreased”, “Yes, increased”, “Yes, much increased”); presence (“Yes”, “No”) and type of chronic disease(s); general aspects of lifestyle and behaviors, including: smoking habit before and during the WH period; eating behavior by assessing changes during WH in the consumption of caffeinated beverages and salty snacks, and in the sense of hunger and satiety (“No”, “Yes, decreased”, “Yes, increased”); adherence to the Mediterranean diet was evaluated using the validated Mediterranean Diet Adherence Score (MEDAS) [29] (see next paragraph for a detailed description). Depression symptoms were assessed through the validated Patient Health Questionnaire-9 (PHQ-9).

The validated questionnaires (MEDAS, PHQ-9) were administered twice, once retrospectively referring to the time before the introduction of WH and once referring to the time after the introduction of WH.

### Questionnaires

MEDAS is a 14-item questionnaire for the assessment of adherence to the traditional Mediterranean diet [29, 30]. The screener includes 14 questions on habitual intakes of several food items. The final MEDAS score can range between 0 and 14, with higher scores indicating a closer resemblance to the Mediterranean diet. For categorization according to the adherence to the Mediterranean diet, we applied the following criteria: low adherence (score ≤5); moderate adherence (score 6–9); and high adherence (score ≥10) [31].

The PHQ-9 [32] is a 9-item index to assess the presence of depression symptoms. The questionnaire assesses how often the subjects had been disturbed by any of the 9 situations on a scale of 0 (not at all), 1 (for several days), 2 (at least half the time), and 3 (nearly every day). The total score ranks severity of depression, i.e. 0–4 (absent), 5–9 (mild), 10–14 (moderate), 15–19 (moderate-severe), and 20–27 (severe).

### Ethical issues

The study was conducted in accordance with the Helsinki Declaration and positively evaluated by the CNR Data Protection Officer. Ethical approval was provided by the CNR Research Ethics and Integrity Committee, on October 28, 2021 (Ethical Clearance number 0078918/2021). Participation was voluntary, without compensation and communication to the CNR administration. All respondents provided written consent, which was a prerequisite for participation, by ticking a check-box before the survey started. Further details are reported in a previous paper [27].

### Statistical analysis

Main survey response data were summarized as counts and percentages for categorical variables, and mean ± standard deviation or median with interquartile interval for continuous variables. The completion of sections including the PHQ-9 and the MEDAS questionnaires was optional, but within each session all questions were mandatory. Furthermore, given that the majority of respondents completed all sections, there was no need to impute missing data. The two-tailed Wilcoxon signed-rank test was used to evaluate pre–versus during WH changes of the scores and the pseudomedian estimate of the location shift was computed. A nonparametric approach was preferred to traditional, Gaussian based, t-test and ANOVA due to the ordinal nature of the scores. Moreover, checks for gaussianity (Shapiro-Wilks test) were rejected more often than not, due to the presence of outliers and asymmetry in the score distributions. For categorical variables, the McNemar test and the Stuart-Maxwell-Test were used to compare discrete distributions before and during the WH period. The bivariate association of habits and changes in lifestyle behaviors and depression with a few socio-demographic and work-related confounding factors were investigated by Wilcoxon test, Kruskall Wallis test with Bonferroni post-hoc correction and chi-square test. Very low frequencies were aggregated with those of neighboring classes for both statistical (chi-squared test) and regulatory reasons, where possible. Otherwise, the low frequencies were not reported, in compliance with the Italian legislation on the protection of personal data (Deontological rules for processing for statistical or scientific research purposes carried out within the National Statistical System, resolution n. 514/2018). Confounding factors were gender, age, macro-region of residence, living alone or not during the COVID-19 pandemic, professional profile, caregiving tasks for cohabiting people, caregiving tasks for non-cohabiting people, home-work commuting time, type of work-room at home, frequency of sharing of the work-room at home.

The 5% significance level was considered. Statistical analysis was performed by R (available online at: https://www.R-project.org/).

Depression symptoms’ worsening/improvement was defined as an increase/decrease of at least one point of PHQ score in the transition to the WH period. Similarly, but in the opposite direction, we proceeded in the case of the MEDAS score.

## Results

### Sample characteristics

A total of 748 subjects participated in the survey and, after validation of the data, all respondents were included in the study. A total of 733 subjects (98%) completed all the sections. General characteristics of the sample are reported in Table 1. Most of the participants were women (57.6%) and in the 40–49 and 50–59 age ranges. The sample showed a high level of education (82.1% graduated or with a higher level of education). At the date of compilation, 61.2% of participants were in the healthy weight range, whereas 29.4% were overweight range, and 7.4% were obese.

**Table 1:** Participants’ characteristics.

	Sample (n = 748)	
	N	%
**Gender**		
Men	317	42.4
Women	431	57.6
**Age (years)**		
≤39	90	12.0
40–49	275	36.8
50–59	285	38.1
≥60	98	13.1
**Cohabitation**		
Living alone	108	14.4
Not living alone, no children	282	37.7
Not living alone, with children	358	47.9
**Italian area** ^**a**^		
North	244	32.6
Center	261	34.9
South	168	22.5
Islands	75	10.0
**Education**		
Graduation	614	82.1
No graduation	134	17.9
**Professional profile**		
Administrative and technical staff	238	31.8
Researcher and Technologist	510	68.2
**BMI**^**b**^ **(kg/m**^**2**^**)** (n = 742)		
Underweight (<18.5 kg/m^2^)	15	2
Normal weight (18.5–24.9 kg/m^2^)	454	61.2
Overweight (25–29.9 kg/m^2^)	218	29.4
Obese (>30 kg/m^2^)	55	7.4
**Comorbidity**	97	12.9

^a^ North: Aosta Valley, Emilia Romagna, Friuli-Venezia Giulia, Liguria, Lombardy, Piedmont, Trentino-Alto Adige, and Veneto. Center: Lazio, Marche, Tuscany, and Umbria. South: Abruzzo, Apulia, Basilicata, Calabria, Campania, Molise. Islands: Sardinia, and Sicily

^b^ BMI: body mass index.

### Main changes in lifestyle behaviors

**Fig 1:**
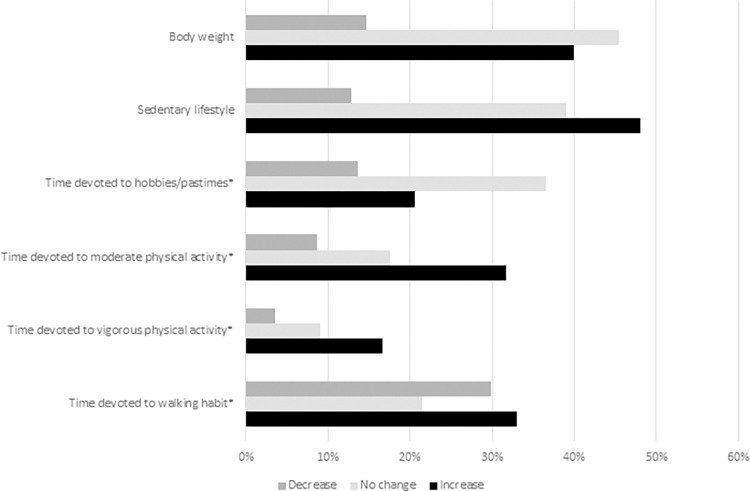
Percentage of participants reporting a decrease, no change, or increase in lifestyle behaviors and body weight during WH compared to before WH. For behaviors marked by *, the sum of the three percentages indicates the frequency of subjects who had the corresponding habit during WH.

We examined answers to questions about habits and changes in lifestyle behaviors during WH. The majority of respondents reported that they were not in the habit of smoking before the COVID-19 pandemic and the introduction of WH did not significantly change this habit (81.1% vs 81.9%, p = 0.24). Changes in lifestyle behaviors and body weight during WH are summarized in Fig 1. Within this general framework, some important variations were observed. The detailed stratified analysis of these changes is reported in S1 Table.

During WH, an increase in sedentary lifestyle was reported by about half of the cases (48.3%). A decreased sedentary habit was more prevalent among women (14%) than men (10%). An increased (even much) sedentary habit was more prevalent among those who lived alone (58%) than among those who did not (46.0%).

Concerning physical activity, a high percentage of subjects, however, were not in the habit of engaging in vigorous (48%) or even moderate (25%) physical activity. 18.6% and 15.1% of subjects reported they did not practice vigorous and moderate physical activity, respectively, during the COVID-19 pandemic because of the containment measures. During the WH period, 58% of subjects practiced moderate activity, and 29% vigorous physical activity, with a large proportion of these subjects reporting an increased time devoted to practicing moderate (55%) or vigorous (43%) physical activity during WH than before the COVID-19 pandemic (including subjects who started practicing during WH). Practicing physical activity was significantly associated with gender (p<0.04): practice during WH was more likely among men (vigorous: 40% vs 21%; moderate: 63% vs 54%), while the lack of the habit was more frequent among women (vigorous 62% vs 39%, moderate: 28% vs 26%). Physical activity was also significantly associated with home-work commuting time (p≤0.04): practicing moderate activity was more frequent in the class of 30–60 minutes than in the other classes (64% vs < 60%). Furthermore, the proportion of subjects who did not practice any activity due to the COVID-19 restrictions was highest among those with the shortest commuting time (moderate: 24% vs < 15%; vigorous: 28% vs < 20%). The lack of habit of moderate physical activity was more frequent among the subjects with the longest home-work commuting time than in the other groups (35% vs < 25%). The reduction in the time dedicated to vigorous physical activity was more frequent among women than men (19% vs 7%), and among subjects who had to share the workroom frequently rather than those who had to share it occasionally or never (24% vs < 11%). As far as the moderate physical activity is concerned, time reduction was more frequent among those who lived alone than among those who did not (27% vs 12%).

The habit of walking for at least 10 minutes was maintained in 84% of cases, of which 39% increased and 35% decreased the time devoted during WH, while 26% reported no changes. Women were less likely than men to keep the dedicated time unchanged (20% vs 34%), and the much time reduction was more frequent among women than men (17% vs 9%). The change in time devoted to walking habit during WH was significantly associated to home-work commuting time. A longer time was in fact more frequent among subjects with the longest commuting time (>60 min) than in the other groups (16% vs < 10%). Moreover, 40% of respondents reported an increase (even a lot) in body weight, which was more frequent among the participants who frequently had to share the workroom at home than among subjects who had occasionally or even never to share (52% vs < 40%).

Participants were also asked to report changes in their habit of pursuing hobbies and pastimes. The majority of the respondents continued to engage in hobbies or pastimes during the COVID-19 pandemic (71%). This occurred more frequently among subjects younger than 40 years as compared to those of older age (19% vs < 15%). However, younger people were more likely to stop practicing hobbies and pastimes than older people (16% vs < 10%). The absence of any hobby/pastime during any period was more frequent, however, among the elderly (over 50, 76% vs < 70%). Overall, men were more likely to keep the habit of having hobbies and pastimes during the COVID-19 pandemic than women (79% vs 65%). Keeping this habit was less likely among participants with caregiving tasks for people living with them than among subjects without these tasks (58% vs 73%). The move to WH provided an opportunity to spend more time on hobbies and pastimes to just under a third of these subjects, without gender differences. Once again, the commuting time was significantly associated with the variation of leisure time (p≪0.001). A decreased time was more frequently observed among subjects with the shortest commuting time (32% vs < 20%), while an increased time among subjects with the longest commuting time (47% vs < 35%). Increased time was also less frequent among those who had to share frequently the workroom than among those who shared only occasionally or never (20% vs > 30%). The transition to WH resulted in the loss of hobbies and pastimes in 8.2% of cases. Of these, 70% are women. This result was more frequent among subjects with caregiving tasks for people not living with them than for subjects without these tasks (9% vs 5%). Nearly the same number of subjects (8.6%) took the opportunity to start a hobby or pastime. Of these, 70% are women and 72% are subjects living not-alone.

During the WH period, 16% of subjects reported an increased intake of caffeinated beverages, and nearly the same proportion a reduced consumption. An increase in the sense of hunger and in the intake of salty snacks was reported by 32% and 21% of participants, respectively.

**Fig 2:**
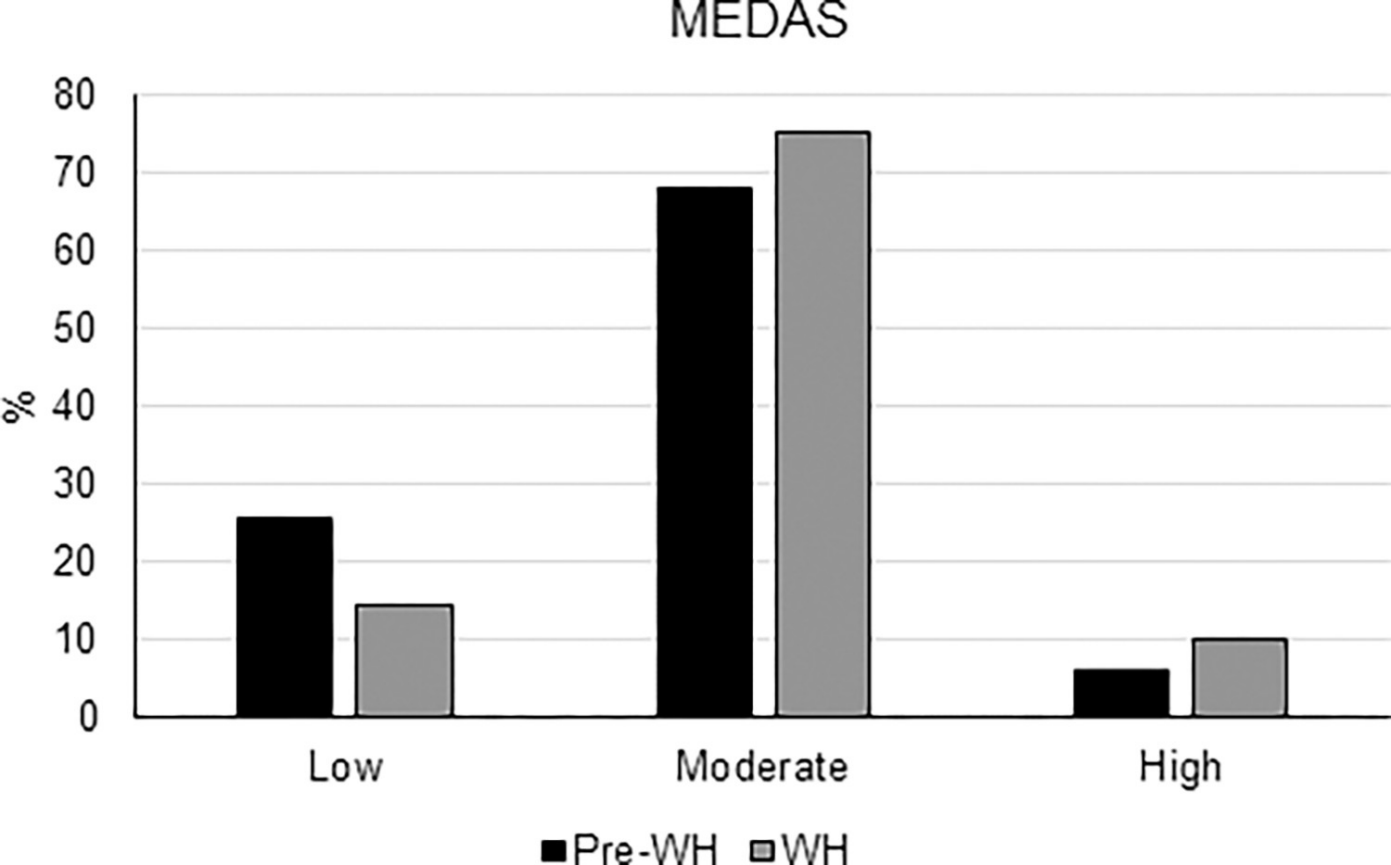
Percentage of participants with low (0–5), moderate (6–9) and high (≥10) adherence to the Mediterranean diet based on MEDAS before WH (pre-WH) and during WH.

Adherence to the Mediterranean diet (MEDAS score) significantly increased during WH compared with before its introduction (7.31 ± 1.75 vs 6.66 ± 1.88; median: 7 vs 7, IQR: 6.0–8.8 vs 5.0–8.0, Wilcoxon test p≪0.001). Of the 742 subjects who answered the section 2, 319 (43%) raised the score by at least 1 point and 148 (20%) by at least 2 points. As shown in Fig 2, the distribution of the subjects into the three classes of low (total score from 0–5), moderate (6–9) and high (≥10) adherence to the Mediterranean diet significantly changed during the WH period compared to before (p≪0.001): the percentages of workers with moderate and high adherence to the Mediterranean diet increased by 7.1% and 4.1%, respectively, during WH compared with pre-WH. A 11.3% decrease of low adherence was in parallel reported.

In general, variations of the adherence were not significantly associated with most of the considered confounding factors. Before WH, a higher adherence to the Mediterranean diet was reported among participants older than 60 years compared with all the other age ranges (p≪0.001). During WH, a higher Mediterranean diet adherence was reported among participants older than 60 years compared with those aged 40–49 years (p<0.02). However, the variation of the MEDAS score was not age-dependent (p = 0.50). Gender difference was also found during WH, with women reporting a higher adherence than men (p<0.003). Again, however, the variation with respect to pre-COVID-19 pandemic did not depend on gender (p = 0.06). Although change in the total score was not significantly associated with the Italian macro-region of residence, a macro-region effect on MEDAS was observed both pre-COVID-19 pandemic and during WH, with greater adherence in the Center macro-region than in the South (p<0.01).

**Fig 3:**
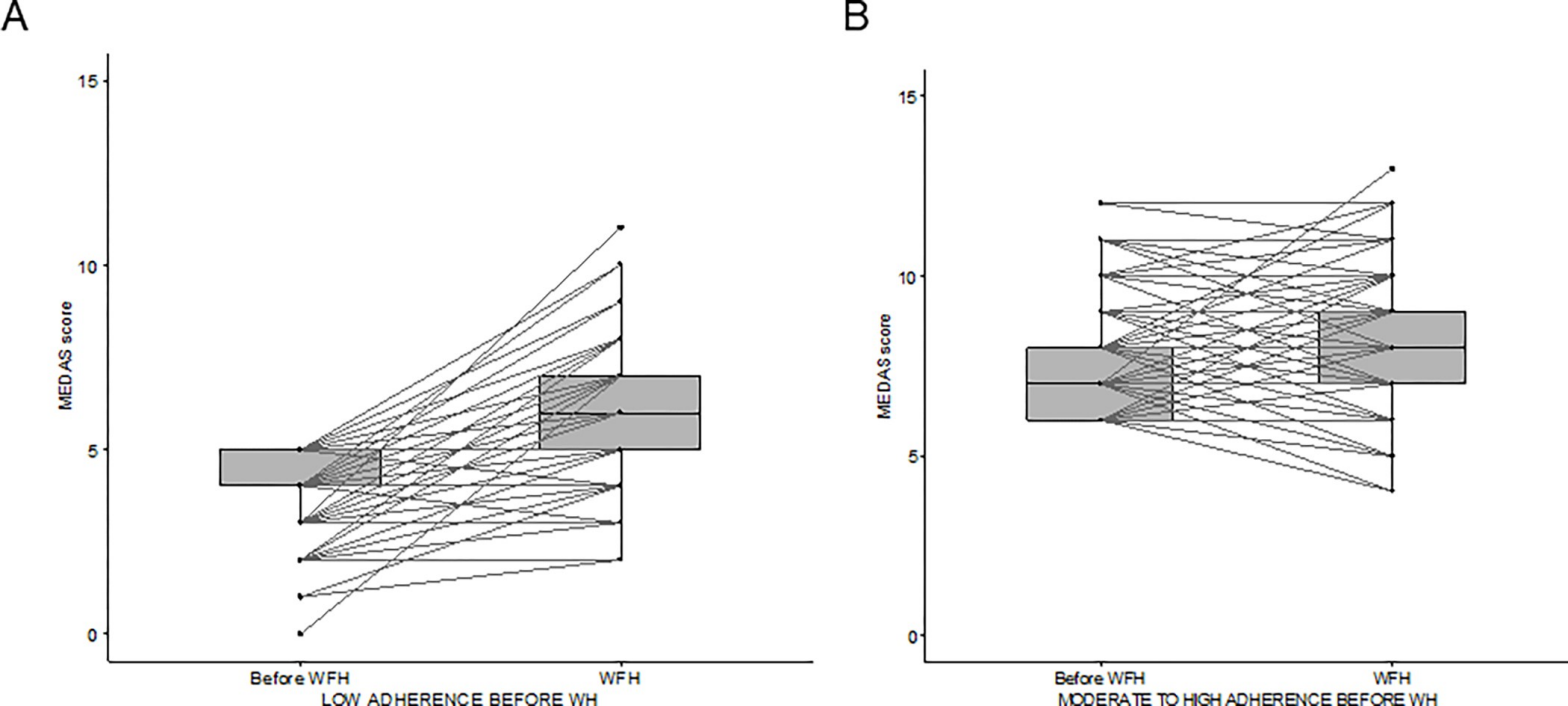
MEDAS score before and during WH in participants reporting low adherence (MEDAS≤5) (A) and moderate to high adherence (MEDAS>5) (B) before the transition to WH.

A preliminary analysis according to the pre-WH adherence to the Mediterranean diet highlighted that both participants with pre-WH low and moderate-high adherence experienced a statistically significant improvement of MEDAS when working from home (p≪0.001) (Fig 3), and that this improvement was significantly higher among those subjects who reported a low adherence before the COVID-19 pandemic (estimated location shift of +2 vs +1, p≪0.001). Moreover, 58% of the subjects with low adherence before the COVID-19 pandemic shifted to a moderate or even high level, while 18% of the participants with at least moderate adherence reduced their score to ≤ 5.

### Changes in depression symptoms

**Fig 4:**
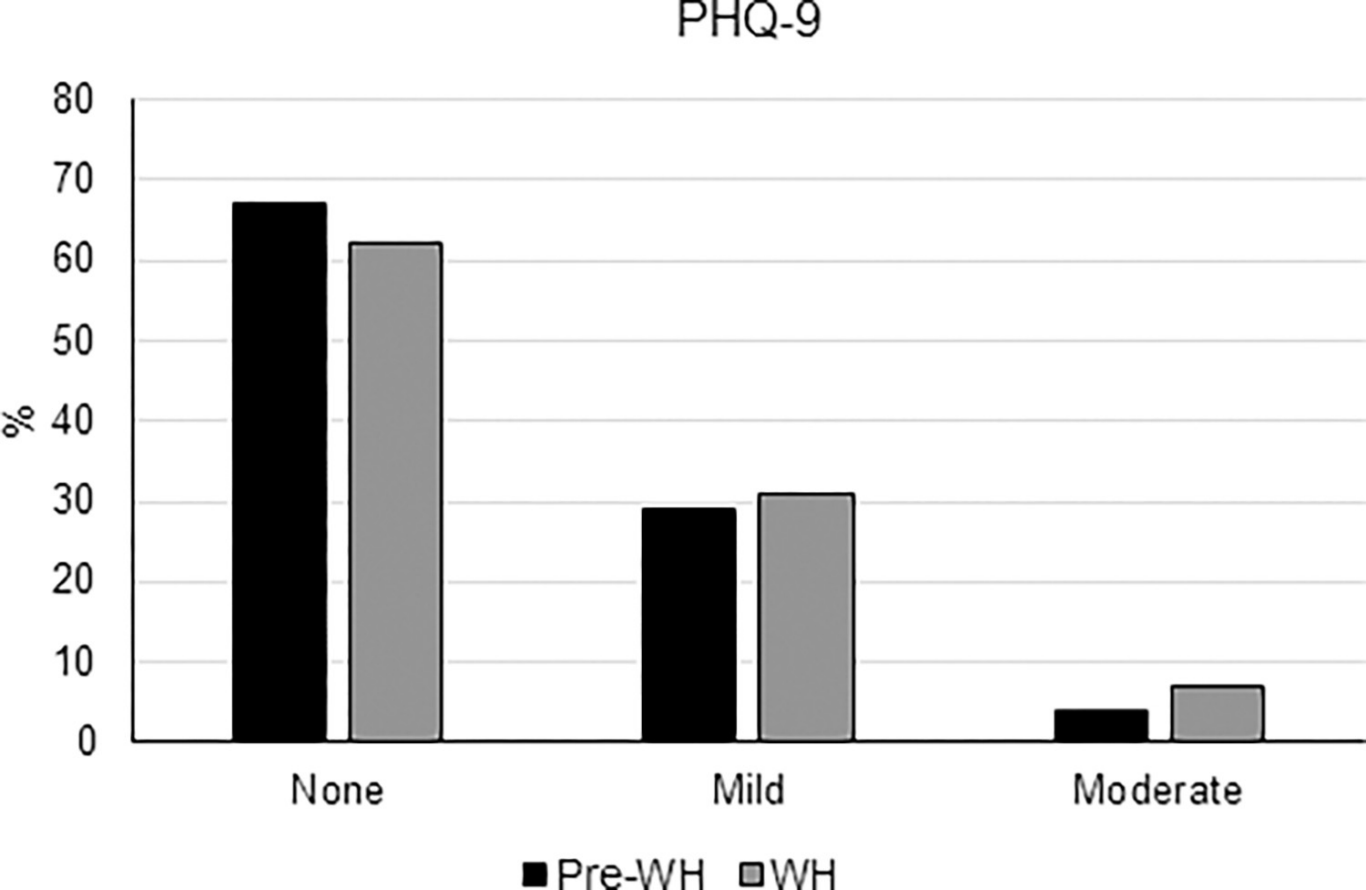
Percentage of participants with none (0–4), mild (5–9) and moderate (≥10) symptoms of depression based on PHQ-9 before WH (pre-WH) and during WH.

The level of depression measured in the entire population using the PHQ-9 score did not vary significantly during the WH experience (4.05 ± 3.39 vs 3.88 ± 3.10; median: 4 vs 3; interquartile range: 2–6 vs 2–5; *p* = 0.09). However, the prevalence of workers with mild symptoms of depression (i.e., a PHQ-9 score from 5–9) increased from 18.1% before WH to 20.1% during the WH period. Similarly, the prevalence of workers with moderate depressive symptoms (i.e., a PHQ-9 score of 10 or more) increased from 4.2% before WH to 6.8% during WH (*p* = 0.006) (Fig 4).

Stratified analysis showed that women reported a higher PHQ-9 score than men both before and during WH (p≪0.001) (S1 Table). However, the location shift did not depend on gender (p = 0.41). Significant results were also obtained for other confounders such as professional profile, with researchers reporting significantly lower total scores than technical or administrative staff (p<0.03), and caregiving, with higher values among those involved in these tasks for people not living with them (p<0.01). As in the case of gender, the variations did not depend significantly on the confounding variable (p>0.45). Age was significantly associated with reported symptoms before the COVID-19 pandemic (p = 0.008), with older subjects (60 or more years) who reported a lower total score than subjects aged 50 to 59 years. Change in total score was significantly associated with marital status (p = 0.03), type of workroom at home (p = 0.03), and frequency of workroom sharing (p = 0.0009). However, only in the latter case estimated differences among groups were different from 0 and then meaningful. The change in the PHQ score was also significantly associated with the commuting time (p = 0.003): subjects with the lowest and the highest commuting time had opposite results, with an increase of the total score among the former and a decrease among the latter. When considering the total score during WH, it was higher among participants who lived alone than among those who did not (p = 0.02). Furthermore, it was lower among those who already had a home office before the COVID-19 pandemic than among other groups of subjects who had to find even temporary solutions during WH (p = 0.002). Finally, it was higher among subjects who had to share the workroom frequently than among those who had to share it occasionally or never (p = 0.02), and among those involved in caregiving tasks for people not living with them (p = 0.006).

**Fig 5:**
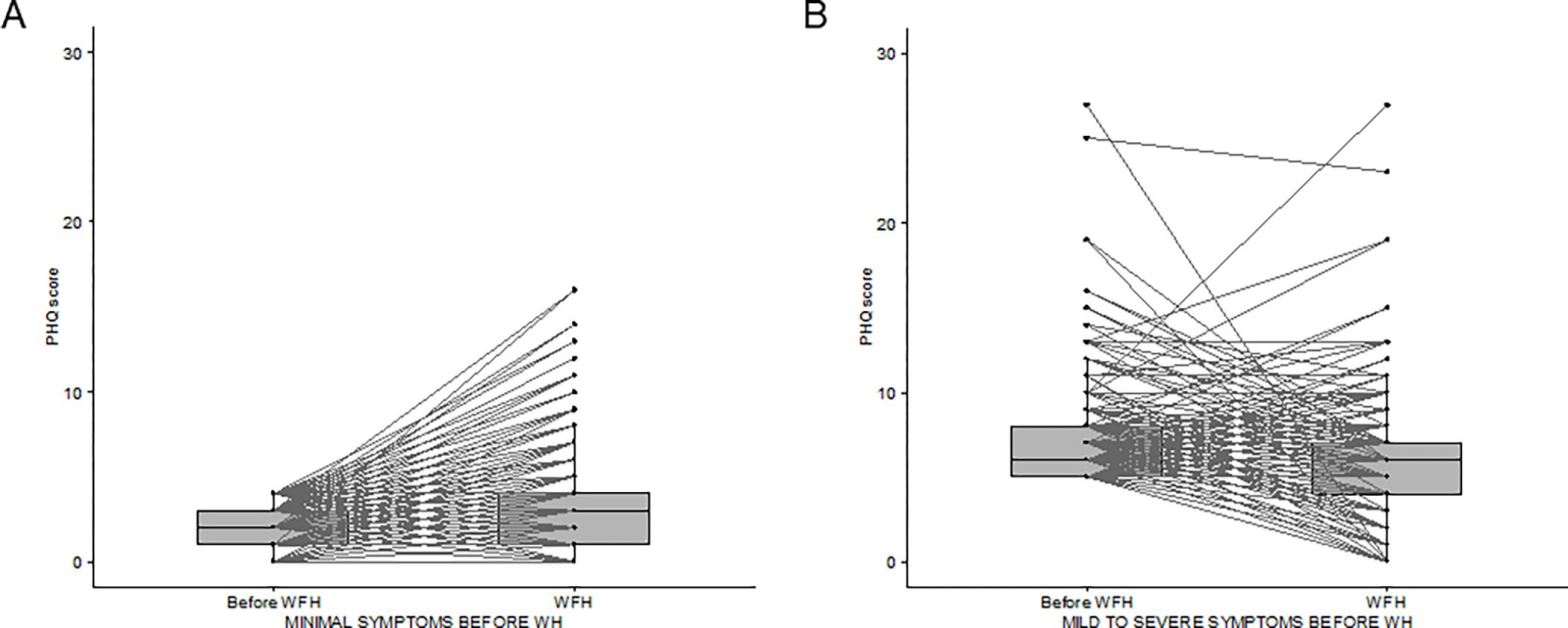
PHQ total score before and during WH in participants reporting minimal depressive symptoms (PHQ ≤ 4) (A) and mild to severe depressive symptoms (PHQ>4) (B) before the transition to WH.

A preliminary analysis according to the pre-COVID-19 pandemic burden of depression symptoms showed that participants with minimal depression symptoms (PHQ ≤ 4) before WH reported a statistically significant worsening of the condition (p≪0.001) with the transition to WH, with an estimated true location shift of the total score of +1 (Fig 5). Of these subjects, 22.6% shifted from minimal to mild or even severe depression during WH. On the contrary, subjects with pre-WH mild to severe depression symptoms (PHQ > 4) reported a statistically significant improvement of the condition (p≪0.001), with an estimated true location shift of the total score of -1.5. Of these respondents, 31.8% reduced their depression score to a minimal level (PHQ ≤ 4).

### Discussion

By conducting an online survey among workers of an Italian public research organization who transitioned to WH due to the COVID-19 pandemic, we examined self-reported changes in selected lifestyle and health-related parameters during WH compared with pre-WH (before the pandemic). Notably, our data were gathered from workers who were experiencing WH after the Italian lockdown period and for almost 18 months after WH implementation in March 2020. Therefore, surveyed workers were increasingly adapted to the WH arrangement in their daily routine during a period of fluctuating but steadily stabilizing COVID-19 epidemiological situation, also thanks to the vaccination campaigns.

Taking this aspect into consideration, we found that among respondents there was an increase in sedentary lifestyle and body weight during WH. However, workers performing moderate-vigorous physical activity before WH increased the time devoted to these activities during WH. Similarly, an increased time devoted to hobbies/pastimes was reported by those already having these habits. We also observed changes in dietary habits during WH with an increased adherence to the healthful Mediterranean diet, but also an increased intake of snacks and a higher hunger sensation. People suffering from depressive symptoms reported a worsening of their mental health during WH.

### Lifestyle behaviors

Solid literature evidence demonstrates that the COVID-19 pandemic severely impacted lifestyle, psychosocial status, and overall health, with heterogeneous magnitude and direction of effects depending on the study population [33]. Most research focused on the effects of the national home confinement(s), which pose important health concerns also in the long-term and especially for comorbid subjects, such as sedentarism, weight gain, reduced physical activity, psychological stress, anxiety and depression, sleep problems and dietary pattern changes [34–36].

Regarding lifestyle behaviors, a decline in physical activity was reported during COVID-19 lockdowns in different countries, including Italy [13, 19, 34, 36–38]. By carrying out a one-year follow-up survey in Italy during the COVID-19 pandemic (from March 2020 to March 2021) in home workers, Rapisarda et al. [19] found that, compared to baseline, physical exercise levels significantly decreased during the lockdown, and increased in the summer, when sports facilities were allowed to reopen, after which a significant decline until the last follow-up was observed. This trend followed the COVID-19 disease waves in Italy. Our results on improved physical activity engagement during WH in terms of time spent among practitioners agree with and extend other few studies reporting similar findings [18, 39, 40], which were limited to home confinement period(s) or conducted after the relaxation of restrictive measures, as in our case.

WH is associated with more time spent at home and thus potentially with more family relationships and environment sharing [41]. Employees save daily commuting time and have the opportunity to set an individualized and flexible approach to work schedule and greater work-life balance, such as choosing to work at times when they feel most productive or comfortable, and/or taking breaks in case of family needs and using additional leisure time, for example to exercise [42]. This may have contributed, as also observed in our study, to an increased availability and use of time for moderate and vigorous physical activity at home or outside when possible, as well as for hobbies and pastimes [43]. This pattern of change can be beneficial for physical and mental health [44, 45]. Furthermore, the COVID-19 pandemic may have prompted and motivated people to be more health-conscious and to adopt healthier lifestyle behaviors, including regular physical exercise, in order to maintain health and strengthen immunity [46, 47]. Supportively, the World Health Organization (WHO) indicated that home confinement should not impede people from engaging in physical activities [48]. Research indeed suggests that some people actually started exercising more during the COVID-19 pandemic, for example by practicing in-home training [42]. Our stratified analysis demonstrated that habit and changes in lifestyle behaviors were different according to sociodemographic and work-related factors. Men were more likely to be physically active then women and to keep the habit of having hobbies and pastimes during the COVID-19 pandemic. Moreover, women were more likely to decrease the time devoted to walking and to vigorous physical activity, and to lose or to start hobbies and pastimes during WH. As expected, a longer commuting time was associated with the lack of (moderate) physical activity habit, but also with an increased time for walking and for hobbies/pastimes during WH in those who usually relied on public or private transport to reach the workplace. Living alone was associated with a decreased time dedicated to (moderate) physical activity. The frequent sharing of the workroom was instead associated with decreased engagement in (vigorous) physical activity and hobbies/pastimes during WH. Caregiving tasks were associated with the loss of hobbies and pastimes with the transition to WH.

Nevertheless, as consistently observed in many previous investigations in the general population or professional cohorts [18, 19, 34, 39, 49], we registered an increase in sedentary behavior during WH, and this was more frequent among those who lived alone and less frequent among women. Workers who used to commute to work, worked in an in-presence setting, and interacted with colleagues face-to-face, could now spend longer hours sitting in front of a computer screen [17]. Exacerbation of sedentary behavior may increase the risk for pain and discomfort as well as for adverse health outcomes [49–52], thus claiming specific attention in order to reduce sitting time in home workers. An increased prevalence of metabolic syndrome was reported for sedentary occupation types [53]. Of note, as observed in the current study, an increase in vigorous and moderate intensity activity among home workers may not necessarily lead to a decrease in sedentary time, because bouts of physical activity during the day might not be a large part of the time, and workers can still be sedentary for most of the day.

Another negative aspect of the forced transition to WH was that almost 40% of participants reported an increase in body weight during WH than before. This is consistent with results from other studies conducted during the lockdown imposed by the COVID-19 pandemic [19, 34, 36, 54, 55]. This finding raises concerns about the potential negative consequences for home workers, as body weight gain is associated with chronic disease risk, such as cardiometabolic disease and cancer [56]. It is interesting to note that the majority of the study participants (61.2%) were in the normal weight range for BMI on the date the questionnaire was completed, while only 7.4% were obese, and 29.4% of participants declared to be overweight, which approaches prevalence data for BMI categories in Italy [57]. Notably, an important proportion of subjects self-reported to be overweight at the compilation date, which might expose them to the risk of developing obesity during the COVID-19 pandemic [54] and to the adverse health outcomes of gaining weight.

In our study, the increased sedentary behavior may have contributed to the reported body weight gain, but unhealthy eating habit may also play a role, including a higher intake of calories and/or of nutrient-poor foods. In accordance with the reported weight gain, an enhanced sense of hunger was perceived by participants during WH than before WH, which was accompanied by a self-reported increased intake of packaged salty snacks. Ultra-processed, hyperpalatable snacks containing added salt, sugar, and fats, low in nutrients and high in calories can add excess nutrient-poor calories [58]. Several reports converge to the observation that under the COVID-19 pandemic unhealthy eating patterns (e.g., eating more, snacking more) could emerge [21, 34, 35, 55, 59–62]. In the study by Di Renzo et al. [55], which was conducted in the general population during the lockdown in Italy, an increase in hunger sensation and night snack consumption was observed, and this was found to be associated with a higher intake of junk food and a higher BMI. Another Japanese study found that WH was associated with an increased frequency of snacking [21], and that junk food intake and overall food intake were associated with more physical and mental health issues during WH [13]. The COVID-19 pandemic and factors related to WH represent stressful and anxious events, which, in conjunction with boredom, can increase food cravings and the intake of tasty foods as a (mal)adaptation strategy (emotional eating) [20, 61, 63]. Furthermore, spending more time at home during WH may increase eating occasions and disclose potential negative dietary habits of the subjects [62].

However, evidence suggests that overall diet quality improved during the COVID-19 pandemic and in particular during WH compared to the pre-pandemic period [18, 21, 60, 64, 65]: it was indeed observed an increased intake of healthful foods including olive oil, fruits, vegetables, legumes, nuts, fish, dairy products, organic and locally grown foods [21, 55, 66], and home-made foods, which tend to be healthier than foods prepared away from home [67].

The degree of adherence to the Mediterranean diet, one of the most healthful dietary patterns, during the COVID-19 pandemic was examined in some studies conducted mostly in Mediterranean countries and a few in non-Mediterranean countries [68]. As summarized in recent systematic reviews [69, 70], results were heterogeneous depending on several potential factors including the methods to assess Mediterranean diet adherence, the use of different thresholds of adherence, and the study countries. In agreement with the systematic review by Della Valle et al. [69], which considered only studies that used validated scales of adherence to the Mediterranean diet, the overall results point to an increased adherence to the Mediterranean diet and/or an increased intake of typical Mediterranean diet foods. In accordance with this background, we here found that: 1) the majority of our participants (68.2%) had a moderate (6–9 points) compliance to the typical Mediterranean diet before the COVID-19 pandemic, and 2) the percentages of workers with moderate (6–9 points) and high (≥10 points) adherence to the Mediterranean diet, based on MEDAS, significantly increased during WH compared with pre-WH. Moreover, an improvement by ≥1 point of MEDAS was observed in 43% of participants. Notably, a greater improvement was found among those subjects who reported a low adherence before the COVID-19 pandemic. Workers with a higher adherence to the Mediterranean diet before and during WH were more likely older than 60 years and to live in Center Italy. During WH, women were more likely to report a higher adherence to the Mediterranean diet. Previous findings also from Italy showed that women and older people displayed a better diet quality, including a higher compliance to the Mediterranean diet and an increased adherence during the confinement period [59, 65, 71, 72]. The observation that Mediterranean diet adherence improved in Italy and other Mediterranean countries during WH in the COVID-19 pandemic [18, 65, 72] is important because those countries are the same where the Mediterranean diet is most popular but is also progressively abandoned in favor of the western dietary pattern since the second half of the 21st century [73]. Our and other studies, as highlighted above, indicate an inversion of the negative trend observed so far in the literature, which could be the results of the pandemic outbreak and related work and life changes.

The availability of more time at home and the higher working schedule flexibility may have allowed more time for cooking, meal planning, family life that might foster healthier dietary patterns [18, 64, 65, 72]. The (functional) fear of contagion [74] during the COVID-19 pandemic could add a positive influence on behavior change, motivating individuals towards the adaptive introduction of healthier habits. But all these hypotheses need to be further verified.

### Depression symptoms

Using the PHQ-9 score to assess the psychological health of our surveyed working population, we found that the sample’s level of mental health did not change during the transition to WH. However, some changes were observed within the population: an increase in the prevalence of mild and moderate depressive symptoms was found, compared to the pre-COVID-19 pandemic period. At the same time, many subjects already presenting mild to severe depression experienced an improvement of their mental health (lower depression score) with the transition to WH.

The COVID-19 pandemic severely impacted mental health, with studies showing increased stress, anxiety, depressive symptoms in the general population [2, 75–80]. This unprecedented situation associated with the emergency led to fear of infection, social isolation, uncertainty about the future, especially in young people and in women, who are generally more likely to suffer from depressive disorder than men [78, 79, 81].

Since the onset of the COVID-19 pandemic, the literature has been flooded with articles reporting the presence of mental disorders in some categories of workers, in particular frontline healthcare workers, and their evolution in relation to the different phases of the pandemic [25, 82, 83]. Notably, the population that was the subject of the present research had no professional exposure to the SARS-CoV-2 virus and, consequently, could be assimilated to office workers. The literature on the psychological impact of transition to WH among these workers during the COVID-19 pandemic reported mixed results, though most studies found a detrimental effect [16]. A decline of mental health status and an increased number of new mental health issues (e.g. anxiety, depression, insomnia or trouble sleeping, mental stress, worry, mood swings, social isolating or decreased interest in social engagement; and trouble concentrating, maintaining attention or focus) were found in workers that were abruptly asked to work from home due to stay-at-home mandates, especially in women [13]. Similar results were found in a Brazilian study where longer working hours during WH in the COVID-19 pandemic context were associated with higher odds of mental health outcomes among women, while displaying a protective effect among men [84]. Another study during the stay-at-home recommendation in Japan found, instead, that starting WH mitigated the negative effect of the COVID-19 pandemic on depressive symptoms [12]. In the LOST in Italy study, subjects who reported reduced housework help from parents during home confinement experienced worsened mental health outcomes, and this association was stronger among those employed at workplace as opposed to those working from home [80], underscoring that the impact of the COVID-19 on mental health might be modulated by work arrangements. It should be noted that most of the previous studies evaluated mental health issues, like other health-related variables, during COVID-19-related containment, which could amplify specific health risks associated with WH. The background influence of the COVID-19 pandemic context is pervasive and should be taken into consideration in interpreting results. Our study is novel in that it was conducted following earlier containment measures and allowed the assessment of specific medium/long-term effects of WH on health-related variables. Another survey conducted among Canadian adults who worked from home during the COVID-19 pandemic found a reduction in the levels of stress and burnout, as well as a decline in general mental health over time [85].

Previous findings showed that mental health could be affected by different factors linked to WH and potentially further burdened by the COVID-19-related context, such as decreased communications with coworkers [13], overload of information and communication technologies [86], concentration deficit [13], absence of a dedicated or adequate workroom at home [13, 87], increased screen exposure and fatigue [88], increased workload and length of workday [13, 89], and work-family conflict mostly for women who may have a greater involvement in household and caregiving tasks than men [13, 90–92]. In line with these findings, we found that higher depressive symptoms both before and during WH were associated with female gender, and with living alone, caregiving tasks during WH, as well as characteristics of the physical workspace such as the frequent sharing of workroom and the lack of a dedicated work office, which may increase distress, distractions and interruptions. Interestingly, lower depressive symptoms before WH were found among older workers (>60 years) and according to job profile, specifically, among researchers compared with technical and administrative staff.

Summarizing, our study in workers of a research organization found that WH during the COVID-19 pandemic was associated with an improved diet quality as indexed by higher adherence to the Mediterranean diet; many workers, however, reported increased snack intake, sedentary habit and body weight gain. Workers performing moderate/vigorous physical activity enhanced this behavior. Literature evidence mostly agrees with the habits and changes we here found in the assayed working population during the COVID-19 pandemic[16], with the difference that our study was conducted several months after the stay-at-home orders in Italy and when barriers to physical mobility were loosened. In accordance with most previous evidence, depression symptoms also increased during WH [16, 93], but those subjects with pre-existing mild to moderate depression reported an improvement. This novel finding would suggest a differential impact of the COVID-19 pandemic on mental health depending on individual vulnerability and adaptation to new contextual factors and to work and life changes.

In agreement with the current literature [16, 94], both positive and negative aspects therefore emerge in the association between WH during the COVID-19 pandemic and measures of health and wellbeing. WH or hybrid working models (work at workplaces some days and working remotely on other days) are expected to be increasingly adopted by companies and governments, and to be mainly on a voluntary basis for workers. This picture has stimulated a reformulation of existing legislation relating to flexible working modalities [95]. It has also raised concerns regarding occupational safety and health risks associated to WH, with undeniable implications for both the economic sector as well as for individual and public health [94]. Further research is needed to build evidence on the impact of WH on workers’ health and to separate out pandemic-specific influences. From our study and the literature evidence available so far, some indications emerge that can be used to develop and implement WH policies to optimize workers’ health and wellbeing and prevent chronic diseases. These would encompass countermeasures at the individual (workers), organizational and societal/governmental levels to mitigate the potential negative effects of WH on workers’ health, including mental health problems and sedentary behavior, as well as interventions to maximize the positive effects, such as healthier lifestyle habits, with a constant addressing of potential health inequalities (e.g., gender, job profile, socioeconomic circumstances).

The study has several limitations. One main limitation is the concurrent presence of the COVID-19 pandemic when workers adopted the WH modality and were here evaluated. It could be therefore plausible that the observed results are driven -or biased- by the pandemic context beside being dependent of specific effects of WH. Therefore, caution should be exercised in interpreting the study results, and further investigations in post-pandemic context are warranted.

The study was conducted in a convenience sample that was non-representative of the population being studied, and participants were recruited by professional emails, which entails a selection bias. Furthermore, participants who chose not to respond may systematically differ from those who did respond. Thus, we cannot exclude the possibility that the sample may not be representative of the population as a whole, and this can lead to an underrepresentation of specific demographics limiting the study generalizability. As for the internal validity, we underscore that the characteristics of the non-responders might be crucial for the study, and their absence could have distorted the true nature of the relationships being investigated. However, this was a large and homogeneous sample of workers fully characterized in terms of sociodemographic and professional data. Furthermore, the study findings cannot be generalizable to other occupational sectors that have different work arrangements and policies, other job profile and population groups, and to other countries due to different sociodemographic, cultural, and occupational structures. Moreover, the survey was conducted almost 2 years after the timely implementation of WH in Italy, which occurred at the beginning of March 2020. This might have introduced a recall bias in participants’ rating of their responses retrospectively, referring to the pre-WH period. Nevertheless, this temporal range could also be seen as a strength of the study because participants might have adapted to WH further on its sudden introduction, thus allowing us to capture data on the investigated variables far from the lockdown period in Italy (from March to May 2020). As already highlighted in other surveys conducted during the COVID-19 pandemic [12, 13, 96, 97], female gender was overrepresented also in our study. Furthermore, we observed a higher response rate in Northern Italy and a lower response rate in Central Italy compared with the regional distribution of the CNR workers. The Northern regions of Italy were more severely impacted by the COVID-19 than Central and Southern Italy, resulting in a greater psychological burden that may have motivated people living there to participate in the survey to a greater extent, as also supposed by others [98]. Although considering the inherent limitations of an online survey with the collection of self-reported information, the methodology adopted to conduct the survey (with email invitation and check for compilation) precluded multiple compilation by the same person or compilation by persons not invited, and allowed pre- and during WH within-subject comparisons.

## Conclusions

We here provided a picture of the main changes of important health-related correlates during WH under the COVID-19 pandemic. The findings highlight improvements in some lifestyle behaviors (time devoted to physical activity, adherence to the Mediterranean diet), but also a worsening of depressive symptoms, body weight, and sedentary habit. However, it is important to evaluate WH-associated changes in health-related correlates considering their pre-COVID-19 pandemic status in the study population. This pattern of changes suggests neither obvious nor unique effects of WH on individuals’ health during the pandemic. Beyond the COVID-19 pandemic, further studies with a longitudinal design and in different working populations and countries will inform on the impact of WH and provide potential avenues for strategies and policies to improve workers’ health and well-being.

## Supporting information

S1 Table Analysis of the statistical association of habits and changes in lifestyle behaviors and depression with sociodemographic and work-related characteristics of the participants. Chi-square test for categorical variable, Wilcoxon test or Kruskal-Wallis test with Bonferroni correction for continuous variables. Very low frequencies were aggregated with those of neighboring classes for both statistical (chi-squared test) and regulatory reasons, where possible. Otherwise, the low frequencies were not reported, in compliance with the Italian legislation on the protection of personal data (Deontological rules for processing for statistical or scientific research purposes carried out within the National Statistical System, resolution n. 514/2018). For the sake of comparison, these aggregations were made in the same way for all the other confounding variables, regardless of the actual number in the specific cells.
(DOCX)
