# Supplementary material for: Effects of working from home on lifestyle behaviors and mental health during the COVID-19 pandemic: A survey study
Source: PLoS One. 2024 Apr 1;19(4):e0300812. doi: 10.1371/journal.pone.0300812 (PMC10984516; doi:10.1371/journal.pone.0300812)
Supplement: S1 Table — Chi-square test for categorical variable, Wilcoxon test or Kruskal-Wallis test with Bonferroni correction for continuous variables. Very low frequencies were aggregated with those of neighboring classes for both statistical (chi-squared test) and regulatory reasons, where possible. Otherwise, the low frequencies were not reported, in compliance with the Italian legislation on the protection of personal data (Deontological rules for processing for statistical or scientific research purposes carried out within the National Statistical System, resolution n. 514/2018). For the sake of comparison, these aggregations were made in the same way for all the other confounding variables, regardless of the actual number in the specific cells. (DOCX) [file pone.0300812.s001.docx]

**S1 Table**. Analysis of the statistical association of habits and changes in lifestyle behaviors and depression with sociodemographic and work-related characteristics of the participants. Chi-square test for categorical variable, Wilcoxon test or Kruskal-Wallis test with Bonferroni correction for continuous variables. Very low frequencies were aggregated with those of neighboring classes for both statistical (chi-squared test) and regulatory reasons, where possible. Otherwise, the low frequencies were not reported, in compliance with the Italian legislation on the protection of personal data (Deontological rules for processing for statistical or scientific research purposes carried out within the National Statistical System, resolution n. 514/2018). For the sake of comparison, these aggregations were made in the same way for all the other confounding variables, regardless of the actual number in the specific cells.

| **GENDER** | **Men** | **Women** | ***p*-value** |
| --- | --- | --- | --- |
| **Changes in hobbies and pastimes during WH** | | | |
| I maintained the habit of h/p during the WH | 251*^+^ | 279*^-^ | < 0.001 |
| I had h/p and now I have none | 18*^-^ | 43*^+^ |  |
| I had no h/p and now I have | 19*^-^ | 45*^+^ |  |
| I didn't have h/p and I don't have any | 29*^-^ | 64*^+^ |  |
| **Changes in the time devoted to hobbies and pastimes during WH** | | | |
| Decrased, even much | 43 | 59 | 0.50 |
| Unchanged | 134 | 140 |  |
| Increased, even much | 74 | 80 |  |
| **Regular practice of vigorous physical activity during WH** | | | |
| Yes | 128*^+^ | 91*^-^ | $\ll0.001$ |
| Either no due to health conditions or I'm not used to it | 116*^-^ | 244*^+^ |  |
| No, due to COVID restrictions | 71 | 96 |  |
| **Changes in the time devoted to vigorous physical activity during WH** | | | |
| Decreased, even much | 9*^-^ | 17*^+^ | 0.02 |
| Unchanged | 45 | 23 |  |
| Increased | 62 | 42 |  |
| Much increased | 12 | 9 |  |
| **Regular practice of moderate physical activity during WH** | | | |
| Yes | 199*^+^ | 234*^-^ | 0.03 |
| Either no due to health conditions or I'm not used to it | 81*^-^ | 121*^+^ |  |
| No, due to COVID restrictions | 37 | 76 |  |
| **Changes in the time devoted to moderate physical activity during WH** | | | |
| Decrased, even much | 27 | 37 | 0.65 |
| Unchanged | 65 | 67 |  |
| Increased | 90 | 114 |  |
| Much increased | 17 | 16 |  |
| **Habit of walking** | | | |
| Yes | 265 | 366 | 0.70 |
| Either No or No, due to health conditions | 25 | 65 |  |
| **Changes in the time devoted to walking** | | | |
| Much decreased | 23*^-^ | 62*^+^ | <0.001 |
| Decrased | 58 | 80 |  |
| Unchanged | 89*^+^ | 72*^-^ |  |
| Increased | 80 | 116 |  |
| Much increased | 15 | 36 |  |
| **Changes in the time usually spent sitting or lying down during the day (including work)** | | | |
| Decrased, even much | 32*^-3^ | 64*^+3^ | 0.005 |
| Unchanged | 144*^+^ | 148*^-^ |  |
| Increased | 117 | 159 |  |
| Much increased | 24 | 60 |  |
| **Changes in body weight^1^** | | | |
| No | 156 | 181 | 0.13 |
| Decreased | 44 | 65 |  |
| Increased, even much | 114 | 182 |  |
| **MEDAS^1,2^** | | | |
| Pre-pandemic | 6.5 (5, 8) | 7 (5.75, 8) | 0.15 |
| During WH | 7 (6, 8) | 7 (6, 9) | 0.003 |
| Change during WH w.r.t. pre-pandemic | 0 (0, 1) | 0, (0, 1) | 0.06 |
| **PHQ**^3,2^ | | | |
| Pre-pandemic | 3 (1, 5) | 4 (2, 6) | $\ll0.001$ |
| During WH | 3 (1, 5) | 4 (2, 6) | $\ll0.001$ |
| Change during WH w.r.t. pre-pandemic | -2 (-4, 0) | -1 (-3, 1) | 0.41 |
| *^+^ Significantly higher than expected, z-test  *^-^ Significantly lower than expected, z-test  ^1^ 742 respondents  ^2^ Median and inter-quartile interval  ^3^ 740 respondents  w.r.t.: with respect to | | | |

| **AGE** | **≤39 ys** | **40-49** | **50-59** | **≥60 ys** | ***p*-value** |
| --- | --- | --- | --- | --- | --- |
| **Changes in hobbies and pastimes during WH** | | | | | |
| I maintained the habit of h/p  during the WH | 17*^+^ | 36 | 40 | *aggregated*  *to the*  *previous*  *age class* | 0.006 |
| I had h/p and now I have none | 14*^+^ | 24 | 26 |  |  |
| I had no h/p and now I have | 10 | 25 | 26 |  |  |
| I didn't have h/p and I don't have any | 49*^-^ | 190 | 291*^+^ |  |  |
| **Changes in the time devoted to hobbies and pastimes during WH** | | | | | |
| Decreased, even much | 5 | 32 | 49 | 16 | 0.31 |
| Unchanged | 26 | 102 | 99 | 47 |  |
| Increased, even much | 18 | 56 | 58 | 22 |  |
| **Regular practice of vigorous physical activity during WH** | | | | | |
| Yes | 40 | 86 | 74*^+^ | 19 | 0.008 |
| Either no due to health conditions  or I'm not used to it | 38*^-^ | 139 | 147 | 66*^+^ |  |
| No, due to COVID restrictions | 12*^+^ | 50 | 64 | 13*^-^ |  |
| **Changes in the time devoted to vigorous physical activity during WH** | | | | | |
| Decreased, even much | *aggregated*  *to the*  *next*  *age class* | 15 | 11 | *aggregated*  *to the*  *previous*  *age class* | 0.047 |
| Unchanged |  | 31*^-^ | 37*^+^ |  |  |
| Increased, even much |  | 80*^+^ | 45*^-^ |  |  |
| **Regular practice of moderate physical activity during WH** | | | | | |
| Yes | 58 | 161 | 155 | 59 | 0.20 |
| Either no due to health conditions  or I'm not used to it | 25 | 76 | 75 | 26 |  |
| No, due to COVID restrictions | 7 | 38 | 55 | 13 |  |
| **Changes in the time devoted to moderate physical activity during WH** | | | | | |
| Decreased, even much | *aggregated*  *to the*  *next*  *age class* | 27 | 25 | 12 | 0.28 |
| Unchanged |  | 62 | 50 | 20 |  |
| Increased, even much |  | 130 | 80 | 27 |  |
| **Habit of walking** | | | | | |
| Yes | 73 | 227 | 244 | 87 | 0.36 |
| Either No or  No, due to health conditions | 17 | 48 | 41 | 11 |  |
| **Changes in the time devoted to walking** | | | | | |
| Much decreased | 7 | 32 | 34 | 12 | 0.62 |
| Decrased | 16 | 41 | 64 | 17 |  |
| Unchanged | 17 | 63 | 56 | 25 |  |
| Increased | 24 | 75 | 69 | 28 |  |
| Much increased | 9 | 16 | 21 | 5 |  |
| **Changes in the time usually spent sitting or lying down during the day (including work)** | | | | | |
| Decreased, even much | 19 | 37 | 29 | 11 | 0.24 |
| Unchanged | 30 | 111 | 109 | 42 |  |
| Increased | 34 | 100 | 107 | 35 |  |
| Much increased | 7 | 27 | 40 | 10 |  |
| **Changes in body weight^1^** | | | | | |
| No | 42 | 116 | 134 | 45 | 0.12 |
| Decreased | 20 | 45 | 34 | 10 |  |
| Increased, even much | 27 | 113 | 114 | 42 |  |
| **MEDAS^1,2^** |  |  |  |  |  |
| Pre-pandemic | 6 (5,8) | 6 (5,8) | 7 (5,8) | 8 (6,8) | $\ll0.001$^4^ |
| During WH | 7 (6,8) | 7 (6,8) | 7 (6,8) | 8 (7,9) | 0.02^5^ |
| Change during WH w.r.t.  pre-pandemic | 0 (0,2) | 0 (0,1) | 0 (0,1) | 0 (0,1) | 0.50 |
| **PHQ^3,2^** |  |  |  |  |  |
| Pre-pandemic | 4 (2,5) | 3 (2,5) | 4 (2,5) | 3 (1,4) | 0.008^6^ |
| During WH | 4 (2,6) | 3 (2,6) | 4 (2,6) | 3 (1,5) | 0.11 |
| Change during WH w.r.t.  pre-pandemic | 0 (-1.25, 1-25) | 0 (-1,2) | 0 (-1,1) | 0 (-0.75, 1) | 0.44 |
| *^+^ Significantly higher than expected, z-test  *^-^ Significantly lower than expected, z-test  ^1^ 742 respondents  ^2^ Median and inter-quartile interval  ^3^ 740 respondents  ^4^ Among the older ones, adherence is significantly higher than in each of the other age groups  ^5^ Among older people, adherence is significantly higher than in the 40-49 age group  ^6^ Among older adults, symptoms of depression are significantly lower than in the 50-59 age group  w.r.t.: with respect to | | | | | |

| **living alone or not during the pandemic** | **Not alone** | **Alone** | ***p*-value** |
| --- | --- | --- | --- |
| **Changes in hobbies and pastimes during WH** | | | |
| I maintained the habit of h/p during the WH | 84 | 9 | 0.003 |
| I had h/p and now I have none | 60 | 4 |  |
| I had no h/p and now I have | 44*^-^ | 17*^+^ |  |
| I didn't have h/p and I don't have any | 452 | 78 |  |
| **Changes in the time devoted to hobbies and pastimes during WH** | | | |
| Decreased, even much | 90 | 12 | 0.42 |
| Unchanged | 235 | 39 |  |
| Increased, even much | 127 | 27 |  |
| **Regular practice of vigorous physical activity during WH** | | | |
| Yes | 187 | 32 | 0.96 |
| Either no due to health conditions  or I'm not used to it | 335 | 55 |  |
| No, due to COVID restrictions | 118 | 21 |  |
| **Changes in the time devoted to vigorous physical activity during WH** | | | |
| Decreased, even much | 19 | 7 | 0.13 |
| Unchanged | 61 | 7 |  |
| Increased, even much | 107 | 18 |  |
| **Regular practice of moderate physical activity during WH** | | | |
| Yes | 364 | 69 | 0.31 |
| Either no due to health conditions  or I'm not used to it | 179 | 23 |  |
| No, due to COVID restrictions | 97 | 16 |  |
| **Changes in the time devoted to moderate physical activity during WH** | | | |
| Decreased, even much | 45*^-^ | 19*^+^ | 0.005 |
| Unchanged | 114 | 18 |  |
| Increased, even much | 205 | 32 |  |
| **Habit of walking** | | | |
| Yes | 535 | 96 | 0.21 |
| Either No or No, due to health conditions | 105 | 12 |  |
| **Changes in the time devoted to walking** | | | |
| Much decreased | 65 | 20 | 0.26 |
| Decrased | 119 | 19 |  |
| Unchanged | 139 | 22 |  |
| Increased | 168 | 28 |  |
| Much increased | 44 | 7 |  |
| **Changes in the time usually spent sitting or lying down during the day (including work)** | | | |
| Decreased, even much | 58 | 11 | 0.03 |
| Unchanged | 258 | 34 |  |
| Increased | 233 | 43 |  |
| Much increased | 64*^-^ | 20*^+^ |  |
| **Changes in body weight^1^** | | | |
| No | 292 | 45 | 0.71 |
| Decreased | 91 | 18 |  |
| Increased, even much | 253 | 43 |  |
| **MEDAS^1,2^** | | | |
| Pre-pandemic | 7 (5, 8) | 7 (5, 8) | 0.99 |
| During WH | 7 (6, 9) | 7 (6, 8) | 0.50 |
| Change during WH w.r.t. pre-pandemic | 0 (0,1) | 0 (0,1) | 0.71 |
| **PHQ**^3,2^ | | | |
| Pre-pandemic | 3 (2, 5) | 3 (2, 6) | 0.45 |
| During WH | 3 (2, 5) | 4 (2, 7) | 0.02 |
| Change during WH w.r.t. pre-pandemic | 0 (-1, 1) | 0 (-1, 1) | 0.03^4^ |
| *^+^ Significantly higher than expected, z-test  *^-^ Significantly lower than expected, z-test  ^1^ 742 respondents  ^2^ Median and inter-quartile interval  ^3^ 740 respondents  ^4^ The estimated difference is negligible  w.r.t.: with respect to | | | |

| **PROFESSIONAL PROFILE** | **Technical**  **staff** | **Administrat.**  **staff** | **Technologist** | **Researcher** | ***p*-value** |
| --- | --- | --- | --- | --- | --- |
| **Changes in hobbies and pastimes during WH** | | | | | |
| I maintained the habit of h/p during the WH | 111 | 49 | 61 | 309 | 0.54 |
| I had h/p and now I have none | 11 | *Not shown* | | 37 |  |
| I had no h/p and now I have | 16 |  |  | 38 |  |
| I didn't have h/p and I don't have any | 25 | 11 | 8 | 49 |  |
| **Changes in the time devoted to hobbies and pastimes during WH** | | | | | |
| Decreased, even much | 26 | 6 | 11 | 59 | 0.76 |
| Unchanged | 55 | 29 | 33 | 157 |  |
| Increased, even much | 30 | 14 | 17 | 93 |  |
| **Regular practice of vigorous physical activity during WH** | | | | | |
| Yes | 46 | 19 | 29 | 125 | 0.47 |
| Either no due to health conditions or I'm not used to it | 80 | 43 | 36 | 231 |  |
| No, due to COVID restrictions | 37 | 13 | 12 | 77 |  |
| **Changes in the time devoted to vigorous physical activity during WH** | | | | | |
| Decreased, even much | *Not shown* | | | | 0.41 |
| Unchanged | 13 | 6 | 8 | 41 |  |
| Increased, even much | 29 | 8 | 19 | 69 |  |
| **Regular practice of moderate physical activity during WH** | | | | | |
| Yes | 89 | 38 | 51 | 255 | 0.57 |
| Either no due to health conditions or I'm not used to it | 48 | 24 | 16 | 114 |  |
| No, due to COVID restrictions | 26 | 13 | 10 | 64 |  |
| **Changes in the time devoted to moderate physical activity during WH** | | | | | |
| Decreased, even much | 15 | 5 | 4 | 37 | 0.73 |
| Unchanged | 26 | 16 | 17 | 77 |  |
| Increased, even much | 48 | 17 | 31 | 141 |  |
| **Habit of walking** | | | | | |
| Yes | 131 | 63 | 68 | 369 | 0.37 |
| Either No or No, due to health conditions | 32 | 12 | 9 | 64 |  |
| **Changes in the time devoted to walking** | | | | | |
| Decreased, even much | 46 | 18 | 24 | 135 | 0.64 |
| Unchanged | 33 | 14 | 21 | 93 |  |
| Increased, even much | 52 | 31 | 23 | 141 |  |
| **Changes in the time usually spent sitting or lying down during the day (including work)** | | | | | |
| Decreased, even much | 21 | 11 | 9 | 55 | 0.55 |
| Unchanged | 68 | 31 | 27 | 166 |  |
| Increased | 49 | 25 | 33 | 169 |  |
| Much increased | 25 | 8 | 8 | 43 |  |
| **Changes in body weight^1^** | | | | | |
| No | 76 | 34 | 36 | 191 | 0.86 |
| Decreased | 22 | 14 | 8 | 65 |  |
| Increased, even much | 64 | 27 | 32 | 173 |  |
| **MEDAS^1,2^** | | | | | |
| Pre-pandemic | 6 (5, 8) | 6 (5, 8) | 7 (5, 8) | 7 (6, 8) | 0.11 |
| During WH | 7 (6, 8) | 7 (6, 8) | 7 (6, 8) | 7 (6, 9) | 0.19 |
| Change during WH w.r.t. pre-pandemic | 0 (0,1) | 1 (0, 1) | 0 (0,1) | 0 (0,1) | 0.23 |
| **PHQ**^3,2^ | | | | | |
| Pre-pandemic | 4 (2, 6) | 4 (2, 6) | 3 (2, 5) | 3 (2, 5) | 0.007^4^ |
| During WH | 4 (1, 6) | 5 (2, 7) | 4 (2, 5) | 3 (2, 5) | 0.02^5^ |
| Change during WH w.r.t. pre-pandemic | 0 (-2, 1) | 0 (-1, 2) | 0 (-1, 1) | 0 (-1, 1) | 0.78 |
| *^+^ Significantly higher than expected, z-test  *^-^ Significantly lower than expected, z-test  ^1^ 742 respondents  ^2^ Median and inter-quartile interval  ^3^ 740 respondents  ^4^ The total score of Researchers is significantly lower than the total score of both Technical and Administrative staff. The estimated difference is of -1.  ^5^ The total score of Researchers is significantly lower than the total score of the Administrative staff. The estimated difference is of -1.  w.r.t.: with respect to | | | | | |

| **caregiving tasks for cohabiting people** | **No** | **Yes** | ***p*-value** |
| --- | --- | --- | --- |
| **Changes in hobbies and pastimes during WH** | | | |
| I maintained the habit of h/p during the WH | 469*^-^ | 61*^+^ | 0.004 |
| I had h/p and now I have none | 53 | 8 |  |
| I had no h/p and now I have | 50 | 14 |  |
| I didn't have h/p and I don't have any | 71*^+^ | 22*^-^ |  |
| **Changes in the time devoted to hobbies and pastimes during WH** | | | |
| Decreased | 93 | 9 | 0.37 |
| Unchanged | 244 | 30 |  |
| Increased | 132 | 22 |  |
| **Regular practice of vigorous physical activity during WH** | | | |
| Yes | 192 | 27 | 0.20 |
| Either no due to health conditions  or I'm not used to it | 338 | 52 |  |
| No, due to COVID restrictions | 113 | 26 |  |
| **Changes in the time devoted to vigorous physical activity during WH** | | | |
| Decreeased, even much | 21 | 5 | 0.42 |
| Unchanged | 62 | 6 |  |
| Increased, even much | 109 | 16 |  |
| **Regular practice of moderate physical activity during WH** | | | |
| Yes | 379 | 54 | 0.35 |
| Either no due to health conditions  or I'm not used to it | 169 | 33 |  |
| No, due to COVID restrictions | 95 | 18 |  |
| **Changes in the time devoted to moderate physical activity during WH** | | | |
| Decreased, even much | 58 | 6 | 0.30 |
| Unchanged | 119 | 13 |  |
| Increased, even much | 202 | 35 |  |
| **Habit of walking** | | | |
| Yes | 542 | 89 | 1.00 |
| Either No or No, due to health conditions | 101 | 16 |  |
| **Changes in the time devoted to walking** | | | |
| Much decreased | 70 | 15 | 0.52 |
| Decreased | 122 | 16 |  |
| Unchanged | 141 | 20 |  |
| Increased | 168 | 28 |  |
| Much increased | 41 | 10 |  |
| **Changes in the time usually spent sitting or lying down during the day (including work)** | | | |
| Decreased | 80 | 16 | 0.14 |
| Unchanged | 250 | 42 |  |
| Increased | 246 | 30 |  |
| Much increased | 67 | 17 |  |
| **Changes in body weight^1^** | | | |
| No | 292 | 45 | 0.56 |
| Decreased | 90 | 49 |  |
| Increased, even much | 255 | 41 |  |
| **MEDAS^1,2^** | | | |
| Pre-pandemic | 7 (6, 8) | 6 (5, 8) | 0.09 |
| During WH | 7 (6, 9) | 7 (6, 8) | 0.26 |
| Change during WH w.r.t. pre-pandemic | 0 (0,1) | 0 (0, 1) | 0.45 |
| **PHQ**^3,2^ | | | |
| Pre-pandemic | 3 (2, 5) | 4 (2, 6) | 0.07 |
| During WH | 4 (2, 6) | 4 (2, 6) | 0.27 |
| Change during WH w.r.t. pre-pandemic | 0 (-1, 1) | 0 (-2, 1) | 0.34 |
| *^+^ Significantly higher than expected, z-test  *^-^ Significantly lower than expected, z-test  ^1^ 742 respondents  ^2^ Median and inter-quartile interval  ^3^ 740 respondents  w.r.t.: with respect to | | | |

| **caregiving tasks for non-cohabiting people** | **No** | **Yes** | ***p*-value** |
| --- | --- | --- | --- |
| **Changes in hobbies and pastimes during WH** | | | |
| I maintained the habit of h/p during the WH | 373 | 157 | 0.03 |
| I had h/p and now I have none | 49*^-^ | 12*^+^ |  |
| I had no h/p and now I have | 36 | 28 |  |
| I didn't have h/p and I don't have any | 66 | 27 |  |
| **Changes in the time devoted to hobbies and pastimes during WH** | | | |
| Decreased, even much | 67 | 35 | 0.41 |
| Unchanged | 199 | 75 |  |
| Increased, even much | 107 | 47 |  |
| **Regular practice of vigorous physical activity during WH** | | | |
| Yes | 154 | 65 | 0.40 |
| Either no due to health conditions  or I'm not used to it | 279 | 111 |  |
| No, due to COVID restrictions | 91 | 48 |  |
| **Changes in the time devoted to vigorous physical activity during WH** | | | |
| Decreased, even much | 18 | 6 | 0.66 |
| Unchanged | 40 | 18 |  |
| Increased, even much | 83 | 31 |  |
| **Regular practice of moderate physical activity during WH** | | | |
| Yes | 305 | 128 | 0.09 |
| Either no due to health conditions  or I'm not used to it | 149 | 53 |  |
| No, due to COVID restrictions | 70*^-^ | 43*^+^ |  |
| **Changes in the time devoted to moderate physical activity during WH** | | | |
| Decreased, even much | 37 | 19 | 0.41 |
| Unchanged | 80 | 33 |  |
| Increased, even much | 153 | 58 |  |
| **Habit of walking** | | | |
| Yes | 439 | 192 | 0.58 |
| Either No or No, due to health conditions | 85 | 32 |  |
| **Changes in the time devoted to walking** | | | |
| Much decreased | 59 | 26 | 0.97 |
| Decreased | 95 | 43 |  |
| Unchanged | 115 | 46 |  |
| Increased | 136 | 60 |  |
| Much increased | 34 | 17 |  |
| **Changes in the time usually spent sitting or lying down during the day (including work)** | | | |
| Decreased, even much | 65 | 31 | 0.13 |
| Unchanged | 210 | 82 |  |
| Increased | 199 | 77 |  |
| Much increased | 50 | 34 |  |
| **Changes in body weight^1^** | | | |
| No | 243 | 94 | 0.41 |
| Decreased | 76 | 33 |  |
| Increased, even much | 199 | 97 |  |
| **MEDAS^1,2^** | | | |
| Pre-pandemic | 7 (5, 8) | 7 (5, 8) | 0.94 |
| During WH | 7 (6, 8) | 7 (6, 9) | 0.57 |
| Change during WH w.r.t. pre-pandemic | 0 (0, 1) | 0 (0, 1) | 0.15 |
| **PHQ**^3,2^ | | | |
| Pre-pandemic | 3 (2, 5) | 4 (2, 6) | 0.003 |
| During WH | 3 (2, 5) | 4 (2, 6) | 0.006 |
| Change during WH w.r.t. pre-pandemic | 0 (-1, 1) | 0 (-1, 1.75) | 0.47 |
| *^+^ Significantly higher than expected, z-test  *^-^ Significantly lower than expected, z-test  ^1^ 742 respondents  ^2^ Median and inter-quartile interval  ^3^ 740 respondents  w.r.t.: with respect to | | | |

| **MACRO-REGION** | **North** | **Centre** | **South** | **Islands** | ***p*-value** |
| --- | --- | --- | --- | --- | --- |
| **Changes in hobbies and pastimes during WH** | | | | | |
| I maintained the habit of h/p during the WH | 179 | 180 | 171 | *aggregated*  *to South* | 0.91 |
| I had h/p and now I have none | 20 | 23 | 18 |  |  |
| I had no h/p and now I have | 17 | 25 | 22 |  |  |
| I didn't have h/p and I don't have any | 28 | 33 | 32 |  |  |
| **Changes in the time devoted to hobbies and pastimes during WH** | | | | | |
| Decreased, even much | 25 | 39 | 38 | *aggregated*  *to South* | 0.23 |
| Unchanged | 96 | 89 | 89 |  |  |
| Increased, even much | 58 | 52 | 44 |  |  |
| **Regular practice of vigorous physical activity during WH** | | | | | |
| Yes | 77 | 76 | 39 | 27 | 0.35 |
| Either no due to health conditions  or I'm not used to it | 126 | 133 | 93 | 38 |  |
| No, due to COVID restrictions | 41 | 52 | 36 | 10 |  |
| **Changes in the time devoted to vigorous physical activity during WH** | | | | | |
| Decreased | 12 | 6 | 8 | *aggregated*  *to South* | 0.31 |
| Unchanged | 19 | 24 | 25 |  |  |
| Increased | 46 | 46 | 33 |  |  |
| **Regular practice of moderate physical activity during WH** | | | | | |
| Yes | 147 | 146 | 95 | 45 | 0.28 |
| Either no due to health conditions  or I'm not used to it | 65 | 78 | 38 | 21 |  |
| No, due to COVID restrictions | 32 | 37 | 35 | 9 |  |
| **Changes in the time devoted to moderate physical activity during WH** | | | | | |
| Decreased, even much | 22 | 23 | 19 | *aggregated*  *to South* | 0.60 |
| Unchanged | 42 | 40 | 50 |  |  |
| Increased, even much | 83 | 83 | 71 |  |  |
| **Habit of walking** | | | | | |
| Yes | 212 | 224 | 138 | 57 | 0.10 |
| Either No or No, due to health conditions | 32 | 37 | 30 | 18 |  |
| **Changes in the time devoted to walking** | | | | | |
| Much decreased | 30 | 26 | 29 | *aggregated*  *to South* | 0.57 |
| Decreased | 44 | 47 | 47 |  |  |
| Unchanged | 53 | 57 | 51 |  |  |
| Increased | 62 | 76 | 58 |  |  |
| Much increased | 23 | 18 | 10 |  |  |
| **Changes in the time usually spent sitting or lying down during the day (including work)** | | | | | |
| Decreased, even much | 30 | 34 | 20 | 12 | 0.85 |
| Unchanged | 89 | 106 | 71 | 26 |  |
| Increased | 95 | 94 | 56 | 31 |  |
| Much increased | 30 | 27 | 21 | 6 |  |
| **Changes in body weight^1^** | | | | | |
| No | 126 | 107 | 68 | 36 | 0.18 |
| Decreased | 33 | 43 | 23 | 10 |  |
| Increased, even much | 83 | 107 | 77 | 29 |  |
| **MEDAS^1,2^** | | | | | |
| Pre-pandemic | 7 (6, 8) | 7 (6, 8) | 6 (5, 8) | 6 (5, 8) | 0.003^4^ |
| During WH | 7 (6, 8) | 8 (6, 9) | 7 (6, 8) | 7 (6, 8) | 0.006^4^ |
| Change during WH w.r.t. pre-pandemic | 0 (0, 1) | 0 (0, 1) | 0 (0, 1) | 0 (0, 1) | 0.27 |
| **PHQ**^3,2^ | | | | | |
| Pre-pandemic | 3 (2, 5) | 3 (2, 5) | 4 (2, 6) | 3.5 (1.25, 6) | 0.50 |
| During WH | 4 (2, 6) | 3 (1, 6) | 4 (2, 5) | 4 (1, 5.75) | 0.38 |
| Change during WH w.r.t. pre-pandemic | 0 (-1, 1) | 0 (-1, 1) | 0 (-1, 1) | 0 (-1, 1) | 0.63 |
| *^+^ Significantly higher than expected, z-test  *^-^ Significantly lower than expected, z-test  ^1^ 742 respondents  ^2^ Median and inter-quartile interval  ^3^ 740 respondents  ^4^  The total score is significantly higher in the Center than in the South  w.r.t.: with respect to | | | | | |

| **HOME-WORK COMMUTING TIME** | **<15 min** | **15-30 min.** | **30-60 min.** | **>60 min** | ***p*-value** |
| --- | --- | --- | --- | --- | --- |
| **Changes in hobbies and pastimes during WH** | | | | | |
| I maintained the habit of h/p during the WH | 91 | 173 | 173 | 93 | 0.11 |
| I had h/p and now I have none | 14 | 14 | 25 | 8 |  |
| I had no h/p and now I have | 12 | 14 | 24 | 14 |  |
| I didn't have h/p and I don't have any | 26 | 29 | 22 | 16 |  |
| **Changes in the time devoted to hobbies and pastimes during WH** | | | | | |
| Decreased, even much | 29*^+^ | 34 | 26 | 13 | $\ll0.001$ |
| Unchanged | 50 | 101*^+^ | 87 | 36*^-^ |  |
| Increased, even much | 12*^-^ | 38*^-^ | 60*^+^ | 44*^+^ |  |
| **Regular practice of vigorous physical activity during WH** | | | | | |
| Yes | 35 | 77 | 70 | 37 | 0.04 |
| Either no due to health conditions  or I'm not used to it | 68 | 113 | 134 | 75 |  |
| No, due to COVID restrictions | 40*^+^ | 40 | 40 | 19 |  |
| **Changes in the time devoted to vigorous physical activity during WH** | | | | | |
| Decreased, even much | 7 | 9 | 10 | aggregated to the  previous class | 0.10 |
| Unchanged | 11 | 30 | 27 |  |  |
| Increased, even much | 17 | 38 | 70*^+^ |  |  |
| **Regular practice of moderate physical activity during WH** | | | | | |
| Yes | 74 | 134 | 156*^+^ | 69 | 0.01 |
| Either no due to health conditions  or I'm not used to it | 35 | 64 | 57 | 46*^+^ |  |
| No, due to COVID restrictions | 34*^+^ | 32 | 31 | 16 |  |
| **Changes in the time devoted to moderate physical activity during WH** | | | | | |
| Decreased, even much | 16 | 20 | 21 | 7 | 0.04 |
| Unchanged | 29 | 43 | 45 | 15 |  |
| Increased, even much | 29*^-^ | 71 | 90 | 47*^+^ |  |
| **Habit of walking** | | | | | |
| Yes | 112*^-^ | 187 | 219*^+^ | 113 | 0.01 |
| Either No or No, due to health conditions | 31*^+^ | 43 | 25*^-^ | 18 |  |
| **Changes in the time devoted to walking** | | | | | |
| Much decreased | 19 | 22 | 32 | 12 | 0.04 |
| Decreased | 28 | 41 | 43 | 26 |  |
| Unchanged | 29 | 53 | 58 | 21 |  |
| Increased | 36 | 71 | 86 | 54 |  |
| Much increased | *not shown* | | | |  |
| **Changes in the time usually spent sitting or lying down during the day (including work)** | | | | | |
| Decreased, even much | 20 | 24 | 27 | 25 | 0.16 |
| Unchanged | 45 | 102 | 97 | 48 |  |
| Increased | 57 | 79 | 95 | 45 |  |
| Much increased | 21*^-^ | 25 | 25 | 13*^+^ |  |
| **Changes in body weight^1^** | | | | | |
| No | 71 | 103 | 107 | 56 | 0.24 |
| Decreased | 12 | 32 | 39 | 26 |  |
| Increased, even much | 60 | 94 | 93 | 49 |  |
| **MEDAS^1,2^** | | | | | |
| Pre-pandemic | 7 (5, 8) | 6 (5, 8) | 7 (6, 8) | 7 (5.5, 8) | 0.30 |
| During WH | 7 (6, 9) | 7 (6, 8) | 7 (6, 8) | 8 (6, 9) | 0.60 |
| Change during WH w.r.t. pre-pandemic | 0 (0, 1) | 0 (0, 1) | 0 (0, 1) | 0 (0, 1) | 0.58 |
| **PHQ**^3,2^ | | | | | |
| Pre-pandemic | 3 (2, 5) | 3 (2, 5) | 4 (2, 6) | 4 (2, 5) | 0.07 |
| During WH | 4 (2, 6) | 3 (2, 5.25) | 4 (2, 6) | 3 (1, 5) | 0.31 |
| Change during WH w.r.t. pre-pandemic | 0 (0, 2) | 0 (-1, 1) | 0 (-1, 2) | 0 (-2, 0) | 0.003^4^ |
| *^+^ Significantly higher than expected, z-test  *^-^ Significantly lower than expected, z-test  ^1^ 742 respondents  ^2^ Median and inter-quartile interval  ^3^ 740 respondents  ^4^ In the group with the least commuting time, the change in the total score is significantly greater than in the group with the longest time, and the estimated difference is +1.  w.r.t.: with respect to | | | | | |

| **type of work-room at home** | **Different**  **rooms, with circumstances** | **Fixed and already**  **used as**  **a studio** | **Fixed and temporarily**  **used as**  **a studio** | **Fixed but not**  **used as**  **a studio** | ***p*-value** |
| --- | --- | --- | --- | --- | --- |
| **Changes in hobbies and pastimes during WH** | | | | | |
| I maintained the habit of h/p during the WH | 131 | 149 | 117 | 133 | 0.82 |
| I had h/p and now I have none | 15 | 12 | 16 | 18 |  |
| I had no h/p and now I have | 14 | 16 | 19 | 15 |  |
| I didn't have h/p and I don't have any | 27 | 24 | 22 | 20 |  |
| **Changes in the time devoted to hobbies and pastimes during WH** | | | | | |
| Decreased, even much | 25 | 23 | 24 | 30 | 0.85 |
| Unchanged | 68 | 79 | 61 | 66 |  |
| Increased, even much | 38 | 47 | 32 | 37 |  |
| **Regular practice of vigorous physical activity during WH** | | | | | |
| Yes | 58 | 60 | 51 | 50 | 0.33 |
| Either no due to health conditions  or I'm not used to it | 101 | 108 | 91 | 90 |  |
| No, due to COVID restrictions | 28 | 33 | 32 | 46 |  |
| **Changes in the time devoted to vigorous physical activity during WH** | | | | | |
| Decreased, even much | *not shown* | 0.16 |  |  |  |
| Unchanged | 16 | 24 | 11 | 17 |  |
| Increased, even much | 34 | 34 | 31 | 26 |  |
| **Regular practice of moderate physical activity during WH** | | | | | |
| Yes | 112 | 115 | 100 | 106 | 0.92 |
| Either no due to health conditions  or I'm not used to it | 51 | 56 | 48 | 47 |  |
| No, due to COVID restrictions | 24 | 30 | 26 | 33 |  |
| **Changes in the time devoted to moderate physical activity during WH** | | | | | |
| Decreased, even much | 13 | 10*^-^ | 20 | 21 | 0.02 |
| Unchanged | 32 | 37 | 23 | 40 |  |
| Increased, even much | 67 | 68 | 57 | 45*^+^ |  |
| **Habit of walking** | | | | | |
| Yes | 158 | 173 | 142 | 158 | 0.68 |
| Either No or No, due to health conditions | 29 | 28 | 32 | 28 |  |
| **Changes in the time devoted to walking** | | | | | |
| Much decreased | 25 | 17 | 15 | 28 | 0.31 |
| Decreased | 28 | 35 | 35 | 40 |  |
| Unchanged | 42 | 49 | 37 | 33 |  |
| Increased | 49 | 58 | 40 | 49 |  |
| Much increased | 14 | 14 | 15 | 8 |  |
| **Changes in the time usually spent sitting or lying down during the day (including work)** | | | | | |
| Decreased, even much | 31 | 25 | 23 | 17 | 0.17 |
| Unchanged | 69 | 89 | 64 | 70 |  |
| Increased | 71 | 63 | 71 | 71 |  |
| Much increased | 16 | 24 | 16 | 28 |  |
| **Changes in body weight^1^** | | | | | |
| No | 81 | 93 | 66 | 97 | 0.06 |
| Decreased | 35 | 25 | 30 | 19 |  |
| Increased, even much | 69 | 81 | 77 | 69 |  |
| **MEDAS^1,2^** | | | | | |
| Pre-pandemic | 7 (6, 8) | 7 (6, 8) | 6 (5, 7) | 7 (5, 8) | 0.02^4^ |
| During WH | 7 (6, 9) | 7 (6, 8.5) | 7 (6, 8) | 7 (6, 9) | 0.24 |
| Change during WH w.r.t. pre-pandemic | 0 (0,1) | 0 (0,1) | 0 (0,1) | 0 (0,1) | 0.55 |
| **PHQ**^3,2^ | | | | | |
| Pre-pandemic | 4 (2, 5) | 3 (2, 5) | 3 (2, 5) | 3.5 (2, 5) | 0.65 |
| During WH | 4 (2, 6) | 3 (1, 5) | 3 (2, 6) | 4 (2, 6) | 0.002^5^ |
| Change during WH w.r.t. pre-pandemic | 0 (-1, 2) | 0 (-1, 1) | 0 (-1, 1) | 0 (-1, 2) | 0.03^6^ |
| *^+^ Significantly higher than expected, z-test  *^-^ Significantly lower than expected, z-test  ^1^ 742 respondents  ^2^ Median and inter-quartile interval  ^3^ 740 respondents  ^4^ In the group that used a temporary study room, the total score is lower than in the group that used a different room depending on the circumstances, and the estimated difference is 1 point.  ^5^ In the group that used a room already used as a studio, the score is lower than in both groups of those who used different rooms depending on the conditions or who used a room not already used as a studio. The estimated difference is, in both cases, 1 point  ^6^ The estimated difference is negligible  w.r.t.: with respect to | | | | | |

| **frequency of sharing of the work-room at home** | **Never** | **Occasionally** | **Often or always** | ***p*-value** |
| --- | --- | --- | --- | --- |
| **Changes in hobbies and pastimes during WH** | | | | |
| I maintained the habit of h/p during the WH | 39 | 26 | 28 | 0.24 |
| I had h/p and now I have none | 24 | 26 | 14 |  |
| I had no h/p and now I have | 25 | 22 | 14 |  |
| I didn't have h/p and I don't have any | 258 | 155 | 117 |  |
| **Changes in the time devoted to hobbies and pastimes during WH** | | | | |
| Decreased, even much | 41 | 25 | 36*^+^ | 0.004 |
| Unchanged | 136 | 80 | 58 |  |
| Increased, even much | 81 | 50 | 23*^-^ |  |
| **Regular practice of vigorous physical activity during WH** | | | | |
| Yes | 99 | 75 | 45 | 0.25 |
| Either no due to health conditions  or I'm not used to it | 200 | 112 | 88 |  |
| No, due to COVID restrictions | 57 | 42 | 40 |  |
| **Changes in the time devoted to vigorous physical activity during WH** | | | | |
| Decreased, even much | 7*^-^ | 8 | 11*^+^ | 0.02 |
| Unchanged | 36 | 19 | 13 |  |
| Increased, even much | 56 | 48 | 21 |  |
| **Regular practice of moderate physical activity during WH** | | | | |
| Yes | 199 | 141 | 93 | 0.18 |
| Either no due to health conditions  or I'm not used to it | 102 | 54 | 46 |  |
| No, due to COVID restrictions | 45 | 34 | 34 |  |
| **Changes in the time devoted to moderate physical activity during WH** | | | | |
| Decreased, even much | 30 | 22 | 12 | 0.91 |
| Unchanged | 64 | 41 | 27 |  |
| Increased, even much | 105 | 78 | 54 |  |
| **Habit of walking** | | | | |
| Yes | 294 | 190 | 147 | 0.79 |
| Either No or No, due to health conditions | 52 | 39 | 26 |  |
| **Changes in the time devoted to walking** | | | | |
| Much decreased | 38 | 19 | 28 | 0.27 |
| Decreased | 58 | 47 | 33 |  |
| Unchanged | 79 | 45 | 37 |  |
| Increased | 91 | 64 | 41 |  |
| Much increased | 28 | 15 | 8 |  |
| **Changes in the time usually spent sitting or lying down during the day (including work)** | | | | |
| Decreased, even much | 41 | 31 | 24 | 0.61 |
| Unchanged | 139 | 87 | 66 |  |
| Increased | 121 | 92 | 63 |  |
| Much increased | 45 | 19 | 20 |  |
| **Changes in body weight^1^** | | | | |
| No | 169 | 114 | 54*^-^ | <0.001 |
| Decreased | 44 | 37 | 28 |  |
| Increased | 130 | 76*^-^ | 90*^+^ |  |
| **MEDAS^1,2^** | | | | |
| Pre-pandemic | 7 (5, 8) | 7 (5, 8) | 7 (6 8) | 0.43 |
| During WH | 7 (6, 8) | 7 (6, 8.5) | 7 (6, 9) | 0.93 |
| Change during WH w.r.t. pre-pandemic | 0 (0, 1) | 0 (0, 1) | 0 (0, 1) | 0.04^4^ |
| **PHQ**^3,2^ | | | | |
| Pre-pandemic | 3 (2, 5) | 4 (2, 5) | 3 (2, 5) | 0.72 |
| During WH | 3 (2, 5) | 3 (1, 5) | 4 (2, 7) | 0.02 |
| Change during WH w.r.t. pre-pandemic | 0 (-1, 1) | 0 (-1, 1) | 0 (0, 2) | <0.001^5^ |
| *^+^ Significantly higher than expected, z-test  *^-^ Significantly lower than expected, z-test  ^1^ 742 respondents  ^2^ Median and inter-quartile interval  ^3^ 740 respondents  ^4^ The estimated difference is negligible  ^5^ In the group who often or always room-shared the change in PHQ is significantly different from the change in the group who occasionally room-shared  w.r.t.: with respect to | | | | |
